# Testicular cancer in twins: a meta-analysis

**DOI:** 10.1038/sj.bjc.6604136

**Published:** 2007-12-11

**Authors:** R E Neale, P Carrière, M F G Murphy, P D Baade

**Affiliations:** 1 Division of Population and Clinical Sciences, Queensland Institute of Medical Research, Post Office Royal Brisbane Hospital, Queensland 4029, Australia; 2 School of Public Health, Queensland University of Technology, O Block, D Wing, Victoria Park Rd, Kelvin Grove, Queensland 4059, Australia; 3 Childhood Cancer Research Group, 57 Woodtstock Rd, Oxford OX26HJ, UK; 4 Viertel Centre for Research in Cancer Control, The Cancer Council Queensland, PO Box 201, Spring Hill, Queensland 4004, Australia

**Keywords:** testicular neoplasms, twinning

## Abstract

In a meta-analysis of testicular cancer in twins, twins had a 30% increased risk (estimate 1.31, 95% CI 1.1–1.6), providing indirect support for the hypothesis that *in utero* hormone variations influence risk of testicular cancer. The summary-estimate for dizygotic twins was 1.3 (1.0–1.7) and for monozygotic or same sex twins 1.4 (1.2–1.8).

Testicular germ-cell cancer is either the most common or second most common cancer in men aged 15–45, depending on country, and rates have been increasing substantially in developed countries over the past few decades (Huyghe *et al*, 2007). Despite attempts to identify risk factors, the aetiology remains unclear. Cryptorchidism, age (Garner *et al*, 2005), contra-lateral disease, and family history (Hemminki and Chen, 2006) are the only established risk factors. Both low and high birth weight may also be associated (Michos *et al*, 2007). Evidence from animal models (Newbold *et al*, 1987), and indirectly from human studies (reviewed in Grotmol *et al*, 2006), indicates that the susceptibility for testicular cancer is probably established *in utero*. The relative importance of environmental factors and genetic factors are not clear, but it is likely that both play an aetiological role (Oosterhuis and Looijenga, 2003). Although the mechanism is uncertain, it is thought that foetal exposure to unusually high oestrogen levels, or an imbalance of oestrogen and androgen levels, might contribute to an increased risk. Examining features of pregnancy, which can affect the levels of these hormones, may help to elucidate the aetiology of this cancer. Twinning is of interest, as twin pregnancies, perhaps particularly with dizygotic twins, result in higher maternal levels of serum oestrogens than with singleton pregnancies.

Several studies have suggested an association between being a twin and testicular cancer, but as both testicular cancer and twinning are rare events, inferences have been limited by a lack of precision in most studies. We therefore aimed to improve the precision of the estimate of testicular cancer risk among twins by carrying out a meta-analysis of previously published studies.

## MATERIALS AND METHODS

Studies published in English between 1961 and 2006 were identified through systematic searches of computerised bibliographic databases (Medline and Embase) using combinations of individual search terms and subject headings. We inspected the reference lists of studies retrieved from these initial searches for previously unidentified studies, which were then located on Medline to identify their subject headings. Further searches were then undertaken using these derived subject headings.

We included all studies that had reported the risk of testicular cancer in twins compared with the general population or a specific single birth control group. Some studies did not provide a risk estimate for all male twins, instead reporting monozygotic and dizygotic twin risks separately (Braun *et al*, 1995; Swerdlow *et al*, 1997; Verkasalo *et al*, 1999), but if a total estimate could be calculated from the data provided, these studies were included.

Analyses were conducted using the meta command in Stata (StataCorp LP, College Station, TX, USA), which tests that the true pooled effect is zero. Tests for heterogeneity were based on the *Q* statistic. Due to the small number of studies, we report on the random effect estimates and include the overall fixed effect estimate for completeness. Sensitivity analyses were conducted by assessing the impact of dropping specific studies on the overall estimated pooled effect. While acknowledging their limited power, we used Begg's test and Egger's test to assess whether there was any evidence of publication bias.

## RESULTS

We identified seven studies, which met our inclusion criteria (Table 1; Braun *et al*, 1995; Westergaard *et al*, 1998; Verkasalo *et al*, 1999; Dieckmann *et al*, 2001; Iversen *et al*, 2001; Hemminki and Chen, 2005; Neale *et al*, 2005). Six were cohort or retrospective cohort studies providing relative risks (RRs) or standardised incidence ratios (SIRs), and one (Dieckmann *et al*, 2001) was a case–control study providing an odds ratio (OR). The number of testicular cancer cases ranged from 4 to 112. Two of these studies were from Sweden and it is likely that the later study has some overlap of study subjects with those in the earlier study.

**Figure 1:**
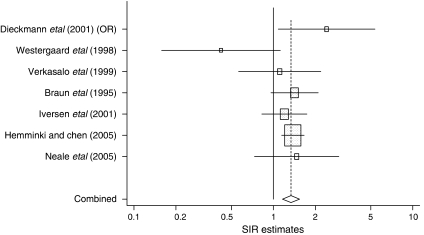
Relative risk of testicular cancer among male twins.

Six of the seven studies reported an increased risk of testicular cancer among twins (Figure 1), with two estimates being significant (Dieckmann *et al*, 2001; Hemminki and Chen, 2005). The smallest study found a non-significant decrease in risk. There was no significant heterogeneity between studies (*Q*=6.18, df=5, *P*=0.289). The pooled estimate of risk was 1.31 (1.1–1.6) assuming random effects and 1.34 (95% CI 1.2–1.5) assuming fixed effects. Excluding the earlier Swedish study resulted in an estimate of 1.3 (1.0–1.7) (*P*=0.068). When we additionally excluded the study with only four cases, we found a risk of testicular cancer among twins of 1.4 (1.2–1.6). We conducted a further analysis excluding the largest and smallest studies, generating a risk estimate of 1.4 (1.1–1.7). Removing the only case–control study did not materially alter the summary estimate. There was no evidence of publication bias (Begg: *z*=−0.45, *P*=0.652; Egger: *t*=−0.63, *P*=0.555).

Five studies either reported the dizygotic twin risk or the risks for male twins that came from an opposite sex twin pair. Pooling these risks resulted in a similar estimate of risks as for all twins (OR 1.3, 95% CI 1.0–1.7). The summary estimate for monozygotic or same sex twins was 1.4 (1.2–1.8).

## DISCUSSION

Only two studies have previously found a significant positive association between twinning and the development of testicular cancer, with four others generating suggestive but non-significant results and one finding a non-significant protective effect. These analyses indicate that these are unlikely to be chance findings, that the result is not sensitive to the inclusion/exclusion of eligible studies, and that there is a real increase in testicular cancer among twins.

Case–control studies have previously suggested a role for maternal and pregnancy-related factors for the development of testicular cancer, with speculation that high *in utero* oestrogen exposure may be the underlying cause (Wanderas *et al*, 1998; Richiardi *et al*, 2002). Twin pregnancies are associated with higher levels of placental hormones than singleton pregnancies, and thus this finding of a significant 30% increase in risk of testicular cancer among twins provides indirect evidence for the *in utero* hormone hypothesis.

Dizygotic mothers have a different menstrual, although not necessarily gestational, hormonal profile than mothers of monozygotic twins. A small proportion of twins will have resulted from ovulation induction in subfertile women who may have distinctly different cyclical hormone patterns, although their pregnancy levels are not likely to be unusual. Some studies included here and others not included report higher testicular cancer risks among dizygotic than monozygotic twins (Braun *et al*, 1995; Swerdlow *et al*, 1997), although this finding is not consistent (Verkasalo *et al*, 1999). Our summary estimates did not support the hypothesis of a higher risk in dizygotic twins than monozygotic or like sex twins, with the estimate for the latter being marginally and not significantly higher. However about half of the like-sex twins would be dizygotic, which could have obscured a real difference.

Apart from the circulating oestrogen hypothesis, there are many other aspects of twin pregnancies (in particular dizygotic pregnancies) that may alter risk of testicular cancer. These include different levels of many other hormones and characteristics of the mother including age, parity, and height (Blondel and Kaminski, 2002). Twins on average weigh about 1000 g less than singleton births and low birth weight may be a risk factor for testicular cancer (Michos *et al*, 2007).

There is emerging evidence that being a twin is protective for childhood cancer, possibly due to the lower birth weight of twins, earlier limitation of their growth velocity, or the selection effects of higher intrauterine death rates (Murphy *et al*, 2001; Neale *et al*, 2005; Milne *et al*, 2007). It is not yet known how far this extends into young adulthood, although it seems likely that there is no long-term difference in the overall risk of cancer in twins compared with singletons (Hemminki and Chen, 2005). Hence any isolated finding in young adulthood, such as that reported here, is salient. These results highlight the importance of exploring maternal factors that influence oestrogen levels, such as obesity, that may be responsible for the rapid increase in testicular cancer incidence that has been seen in many countries over the past several decades.

## Figures and Tables

**Table 1:** Description of studies included in the meta-analysis

**First author, year (country)**	**Number of cases in twins**	**Number of multiple births**	**SIR, RR, or OR (all twins) (95% CI)**	**SIR, RR or OR (DZ or opp. sex twins) (95% CI)**	**SIR, RR, or OR (MZ or same sex twins) (95% CI)**
Braun *et al*, 1995 (Sweden)	25	46 767	1.42 (0.92–2.10)	1.6 (1.0–2.6)	1.1 (0.4–2.2)
Westergaard *et al*, 1998 (Denmark)	4	N/A	0.42 (0.16–1.12)		
Verkasalo *et al*, 1999 (Finland)	8	25 882	1.11 (0.48–2.19)	0.79 (0.22–2.03)	1.87 (0.51–4.78)
Dieckmann *et al*, 2001^a^ (Germany)	14	23	2.41 (1.04–5.63)	2.72 (0.71–11.09)^b^	2.17 (0.62–7.92)^c^
Iversen, 2001 (Norway)	27	233 34	1.20 (0.79–1.74)		
Hemminki and Chen, 2005 (Sweden)	112	139 308	1.38 (1.13–1.66)	1.22 (0.85–1.70)^b^	1.46 (1.15–1.83)^c^
Neale *et al*, 2005 (United States)	14	35 271	1.47 (0.73–2.95)	2.23 (0.58–8.60)^b^	1.11 (0.46–2.63)^c^
Summary estimates	204	270 585	1.3 (1.2–1.5)	1.3 (1.0–1.7)	1.4 (1.2–1.8)

Abbreviations: OR=odds ratio; RR=relative risk; SIR=standardised incidence ratio.

a Case–control study, 418 cases, 636 controls.

b Zygosity determined on basis of being opposite sex.

c Same sex twins–approximately half will be dizygotic.
